# On Hadamard and Kronecker products in covariance structures for genotype × environment interaction

**DOI:** 10.1002/tpg2.20033

**Published:** 2020-07-15

**Authors:** Johannes W. R. Martini, Jose Crossa, Fernando H. Toledo, Jaime Cuevas

**Affiliations:** ^1^ International Maize and Wheat Improvement Center (CIMMYT) Km. 45, El Batán 56237 Texcoco Mexico; ^2^ Universidad de Quintana Roo Del Bosque, 77019 Chetumal, Q.R. Mexico

## Abstract

When including genotype × environment interactions (G × E) in genomic prediction models, Hadamard or Kronecker products have been used to model the covariance structure of interactions. The relation between these two types of modeling has not been made clear in genomic prediction literature. Here, we demonstrate that a certain model based on a Hadamard formulation and another using the Kronecker product lead to exactly the same statistical model. Moreover, we illustrate how a multiplication of entries of covariance matrices is related to modeling locus × environmental‐variable interactions explicitly. Finally, we use a wheat and a maize data set to illustrate that the environmental covariance **E** can be specified easily, also if no information on environmental variables – such as temperature or precipitation – is available. Given that lines have been tested in different environments, the corresponding environmental covariance can simply be estimated from the training set as phenotypic covariance between environments. To achieve a high level of increase in predictive ability, the environmental covariance has to be defined appropriately and records on the performance of the lines of the test set under different environmental conditions have to be included in the training set.

**Abbreviations**
BLUP: best linear unbiased prediction
E1: environment 1
E2: environment 2
E3: environment 3
E4: environment 4
G × E: genotype by environment interaction
GBLUP: genomic best linear unbiased prediction
MET: multi‐environment trials.

## 1 INTRODUCTION

Multi‐environment trials (MET) with which lines are evaluated under several environmental conditions are fundamental in plant breeding. The comparison of their yield‐stability and their genotype × environment interaction (G × E) allows the breeder to select the best genotypes for specific environments or the lines with the most stable yield performance across environments. Early approaches for the analysis of G × E used fixed effects models ignoring that the continuous degree of similarity of environmental conditions at different locations may potentially be incorporated in the statistical model. Similarly, relationship of the lines, as well as spatial trends in the field have not been considered in these early approaches (Cornelius & Crossa, 1999; Cornelius, Crossa, & Seyedsadr, 1996; Crossa & Cornelius, 1997; Cornelius et al., 2001; Crossa et al., 2001). A restriction at that time was the limited availability of appropriate statistical software.

One of the frameworks that easily allows including covariance structures between different random effects are mixed linear models. Mixed linear models and the corresponding best linear unbiased prediction (BLUP) – classically treated by Henderson (1975) – modify the least square regression approach to a penalized regression and provide a framework to incorporate correlations between sites, years, and plots in the field, as well as genetic relation between relatives.

Crossa et al. (2006) illustrated how genetic effects can be modeled as additive and additive × environment interaction using a factor analytic model across environments and the additive pedigree relationship **A**. The authors showed that modeling G × E increases the precision of the predictions. Burgueño, Crossa, Cornelius, Trethowan, McLaren, and Krishnamachari (2007) split the total genetic effects into pedigree based additive and additive × additive effects and modeled the additive × environment interaction and the additive × additive × environment interaction.

With the advances in genotyping methods, dense marker based genomic prediction (Meuwissen, Hayes, & Goddard, 2001) and the selection for predicted genetic values (genomic selection) has become a central component of breeding strategies (Crossa et al., 2011; Crossa et al., 2017; Hayes, Bowman, Chamberlain, & Goddard, 2009; Jannink, Lorenz, & Iwata, 2010). After comparing different types of (genomic) relationship (e.g. Crossa et al., 2010; de los Campos, Gianola, & Rosa, 2009; de los Campos, Gianola, Rosa, Weigel, & Crossa, 2010), a second wave of research has addressed G × E interaction in combination with the genomic relationship matrix **G** (e.g. Burgueño, de los Campos, Weigel, & Crossa, 2012; Lopez‐Cruz et al., 2015; Yao et al., 2017; Gonzales‐Barrios, Diaz‐Garcia, & Gutierrez, 2019).

In some approaches, the covariance structure used as input for the G × E interaction has been modeled using Kronecker products (Cuevas, Crossa, Montesinos‐López, Burgueño, Pérez‐Rodríguez, & de los Campos, 2017), others used Hadamard products (Acosta‐Pech et al., 2017; Basnet et al., 2019; Jarquin et al., 2014; Perez‐Rodriguez et al., 2015; Perez‐Rodriguez et al., 2017; Sukumaran et al., 2017). Moreover, Kronecker products have been used to model the interaction between the parental genomes of hybrids to capture specific combining ability effects (Acosta‐Pech et al., 2017; Basnet et al., 2019). When addressing epistasis, that is genetic interaction, Hadamard products of the additive genomic relationship have mainly been used (e.g. Gao et al., 2017; Jiang & Reif, 2015; Martini, Toledo, & Crossa, 2020; Martini, Wimmer, Erbe, & Simianer, 2016; Varona, Legarra, Toro, & Vitezica, 2018; Vitezica, Legarra, Toro, & Varona, 2017).

**Core Ideas**

- We compare the structure of covariance models for genotype × environment interactions defined by Hadamard or by Kronecker products
- We highlight the identity of modeling marker × environmental‐variable interaction and multiplying covariances
- We present a heuristic approach for defining the environmental covariance

As described, both mathematical operations – the Hadamard and the Kronecker product – have been used to model the covariance of interactions. However, it has not been pointed out what the relation between both types of modeling is and whether and how these approaches differ. In this study, we give a theoretical proof that shows that both methods lead to exactly the same covariance model when used with appropriate design matrices reflecting the arrangement of the phenotypic data. Moreover, we illustrate how multiplying the entries of covariance matrices relates to modeling the interaction between underlying variables, such as markers, temperature or precipitation, explicitly. Finally, we use two publicly available data sets to illustrate that G × E models can improve the prediction accuracy also if no information on the environmental conditions is available. Given that the training set includes a sufficient overlap of lines across environments, estimating the phenotypic covariance from the training set is sufficient to specify the mixed model.

## 2 THEORETICAL BACKGROUND

We will shortly recapitulate the Hadamard, the Kronecker, and the Khatri‐Rao product and illustrate how they can be used to model G × E covariance.

### 2.1 Recapitulation of the Hadamard product

The Hadamard product ○ is defined for two matrices of equal size. Let **A** and **B** be two matrices of size *n* × *m*. Then the Hadamard product A∘B is defined by

$${\left(A\;\circ \;B\right)}_{i,j}:=\;{\left(A\right)}_{i,j}\cdot {\left(B\right)}_{i,j}$$

where *i* = 1,2,…,*n* indicates the row and *j* = 1,2,…,*m* the column. The symbol := means that we define the left hand‐side by the right‐hand side. Moreover, the symbol · denotes the ordinary multiplication of the real numbers. In words, **A** and **B** are multiplied entry‐wise and A∘B is also of size *n* × *m*. Note here that we will be dealing with covariance matrices which are by definition square, that is of size *n* × *n*. Moreover note that A∘B=B∘A.

### 2.2 Recapitulation of the Kronecker product

The Kronecker product ⊗ is defined for any two matrices **A** and **B**, not necessarily of same size. For **A** of size *n* × *m* and **B** of size *k* × *l*,

$$A⊗B:=\left(\begin{array}{ccccc} a_{1,1}B & \;a_{1,2}B & \;\ldots & \;a_{1,\left(m-1\right)}B & \;a_{1,m}B\\ a_{2,1}B & \;a_{2,2}B & \; & \;a_{2,\left(m-1\right)}B & \;a_{2,m}B\\ \vdots & \; & \;\ddots & \;\vdots \\ a_{n,1}B & \;a_{n,2}B & \;\cdots & \;a_{n,\left(m-1\right)}B & \;a_{n,m}B \end{array}\right)$$

Each block $a_{i,j}B$ represents a matrix of size *k* × *l*, which means A⊗B is of size (n·k)×(m·l). For square matrices, their Kronecker product will be square. Note that A⊗B≠B⊗A since the entries of the products will have a different order.

### 2.3 Recapitulation of the Khatri‐Rao product

We recapitulate a third type of product, the column‐wise Kronecker or the Khatri‐Rao product. The Khatri‐Rao product * is defined for any two matrices with the same number of columns. For two matrices **A** of size *n* × *m* and **B** of size *k* × *m*, the *i*‐th column of (A∗B) is defined by

$${\left(A*B\right)}_{•,i}:={\left(A\right)}_{•,i}⊗{\left(B\right)}_{•,i}$$

Consequently, (A∗B) will be of size (n·k)×m. In the case that **A** and **B** are column vectors, the Khatri‐Rao product is identical to the Kronecker product. Note that A∗B≠B∗A since the entries in each column will have a different order.

For more information see for instance Kolda and Bader (2009). In this manuscript, the Khatri‐Rao product will only be relevant for the precise definition of a design matrix in the second theoretical result.

### 2.4 Hadamard and Kronecker products for modeling G × E covariance

Let us assume that we are dealing with three plant lines and phenotypic records in two different environments. The phenotypic data is given by the 6 × 1 column vector

$$y\;={\left(y_{1,1},y_{1,2},y_{2,1},y_{2,2},y_{3,1},y_{3,2}\right)}^{t}$$

where $y_{i,j}$ is the phenotype of the *i*‐th line in the *j*‐th environment. We use the statistical model

(1) $$y\;=\;\mu 1_{6}+Z_{1}g+Z_{2}e+Z_{3}\;\left(\mathrm{ge}\right)+\;\varepsilon$$

where μ denotes a fixed effect, **1**_6_ is a 6 × 1 vector with each entry equal to 1, $g\;∼N(0,\sigma_{g}^{2}\;G)$ the 3 × 1 genetic effects with a covariance (genomic relationship) matrix **G**, and $e\;∼N(0,\sigma_{e}^{2}\;E)$ a 2 × 1 random environmental effect with a covariance **E**. The matrix **E** can be the identity matrix but also a structured covariance obtained for instance from phenotypic correlations across environments or environmental variables (e.g. Perez‐Rodriguez et al., 2015). Moreover, the error is assumed to be $\varepsilon \;∼N(0,\sigma_{\varepsilon }^{2}\;I)$ with identity matrix **I**, meaning that the error terms are independent and identically distributed. The design matrices **Z**_1_, **Z**_2_ and **Z**_3_ are given by

$$Z_{1}=\left(\begin{array}{cc} 1 & \;\begin{array}{cc} 0 & \;0 \end{array}\\ \begin{array}{c} 1\\ \begin{array}{c} 0\\ 0\\ \begin{array}{c} 0\\ 0 \end{array} \end{array} \end{array} & \;\begin{array}{c} \begin{array}{cc} 0 & \;0 \end{array}\\ \begin{array}{c} \begin{array}{cc} 1 & \;0 \end{array}\\ \begin{array}{cc} 1 & \;0 \end{array}\\ \begin{array}{c} \begin{array}{cc} 0 & \;1 \end{array}\\ \begin{array}{cc} 0 & \;1 \end{array} \end{array} \end{array} \end{array} \end{array}\right),\;\;\;Z_{2}=\left(\begin{array}{cc} 1 & \;0\\ \begin{array}{c} 0\\ \begin{array}{c} 1\\ 0\\ \begin{array}{c} 1\\ 0 \end{array} \end{array} \end{array} & \;\begin{array}{c} 1\\ \begin{array}{c} 0\\ 1\\ \begin{array}{c} 0\\ 1 \end{array} \end{array} \end{array} \end{array}\right),\;\;\;Z_{3}=I_{6}$$

assigning the respective genetic effect *i* and environmental effect *j* to the phenotype $y_{i,j}$. We assume the variable “genotype × environment interaction” to be ($\mathrm{ge})\;∼N\left(0,\sigma_{\mathrm{ge}}^{2}\;\mathrm{GE}\right)$ with **GE** being the covariance structure describing the similarity of genotype × environment interactions, and which needs to be specified. Let ${\left(\mathrm{ge}\right)}_{i,j}$ denote the genotype × environment interaction of genotype *i* and environment *j*. As a first approach, we aim at modeling the covariance of ${\left(\mathrm{ge}\right)}_{i,j}$ and ${\left(\mathrm{ge}\right)}_{k,l}$ which should depend on how similar g_*i*_ and g_*k*_ are, as well as on how similar e_*j*_ and e_*l*_ are. A concept that corresponds to using products of underlying variables is to multiply the corresponding covariances:

$$\begin{array}{ccc} \mathrm{COV}\;\left({\left(\mathrm{ge}\right)}_{i,j},{\left(\mathrm{ge}\right)}_{k,l}\right) & = & \;\mathrm{COV}\left(g_{i},g_{k}\right)\cdot \mathrm{COV}\;\left(e_{j},e_{l}\right)\\ & = & G_{i,k}\;\cdot E_{j,l} \end{array}$$

A more detailed formal elaboration on how products of the entries of covariance matrices relate to interactions on a variable effect level can be found in the Appendix. The Kronecker product provides exactly these products of the different entries of **G** and **E**, and thus a first option to define **GE** is

(2) GE:=G⊗E

It is important to highlight that Equation (2) is only valid due to the way the phenotypes are ordered. If we order first according to environment and then according to genotype, we would have to permute **G** and **E** here.

Alternatively, as a second approach, we could say that we do not focus on the covariance of the interaction between **g** and **e**, but rather on the covariance of the interaction between $Z_{1}g$ and$\;Z_{2}e$, that is on the interaction between the two random terms of size 6 × 1. The variance of $Z_{1}g$ is given by $Z_{1}{G\;Z}_{1}^{t}$ and the variance of $Z_{2}e$ is $Z_{2}{\mathrm{EZ}}_{2}^{t}$, both square matrices of size 6 × 6. Instead of using the double index ${\left(\mathrm{ge}\right)}_{k,l}$ for the vector **(ge)** indicating genotype and environment as above, we use here only a single index indicating the position of the considered entry. This means (ge)_*i*_ denotes the *i*‐th entry of vector (**ge**).

Again, as before, we assume that the similarity/covariance of the interaction between ${\left(Z_{1}g\right)}_{i}$ and ${\left(Z_{2}e\right)}_{i}$, which is (ge)_*i*_, and the interaction between ${\left(Z_{1}g\right)}_{j}$ and ${\left(Z_{2}e\right)}_{j}$, which is (ge)_*j*_, depends on the covariances $\mathrm{COV}({\left(Z_{1}g\right)}_{i},{\left(Z_{1}g\right)}_{j})$ and $\mathrm{COV}({\left(Z_{2}e\right)}_{i},{\left(Z_{2}e\right)}_{j})$ and can be modelled as the product:

$$\begin{array}{ccc} \mathrm{COV}\left({\left(\mathrm{ge}\right)}_{i},{\left(\mathrm{ge}\right)}_{j}\right) & = & \mathrm{COV}\left({\left(Z_{1}g\right)}_{i},{\left(Z_{1}g\right)}_{j}\right)\cdot \\ \mathrm{COV}\left({\left(Z_{2}e\right)}_{i},{\left(Z_{2}e\right)}_{j}\right) & = & {\left(Z_{1}{G\;Z}_{1}^{t}\right)}_{i,j}\cdot {\left(Z_{2}{\mathrm{EZ}}_{2}^{t}\right)}_{i,j} \end{array}$$

Thus, we can define the covariance of their interactions as

(3) $$\mathrm{GE}:=Z_{1}{G\;Z}_{1}^{t}\;\circ \;Z_{2}{\mathrm{EZ}}_{2}^{t}$$

Equation (3) is what is most commonly used in literature addressing the topic of G × E (e.g. Jarquin et al., 2014). As we will see in the theoretical results, both operations lead to the same model, that is Equation (2) is equal to Equation (3).

## 3 THEORETICAL RESULTS

We start with two theoretical results.

### 3.1 First theoretical result

Let us consider a data set of *n* lines whose performances were measured in *m* environments. We include each combination of line and environment in the statistical model. Moreover, let ybe a vector of phenotypes ordered primarily according to the genotypes and secondarily according to the environments the record was taken in. Also, let the design matrices, as described above, map the genetic value and the environment to the corresponding phenotype. Then

(4) $$G⊗E\;=\;Z_{1}{G\;Z}_{1}^{t}\;\circ \;Z_{2}{\mathrm{EZ}}_{2}^{t}$$

which means both methods give the same covariance structure **GE**.

We found a similar statement in literature, in which permutation matrices are applied to the Kronecker product on the left‐hand side of the equation leading to a Hadamard product on the right‐hand site (e.g. Liu & Trenkler, 2008; Visick, 2000). However, we have not found Equation (4) in publications addressing the relation between Hadamard and Kronecker products.

#### 3.1.1 Proof of the first theoretical result

This result is a special case of the second theoretical result, which can be found below. Nevertheless, we give a separate proof for both results. In particular, the proof of the second result uses a presentation, which we introduce in the proof of the first result. Both proofs use similar arguments in which the indices are compared and entries are counted.

Let us consider the entry ${\left(G⊗E\right)}_{p,q}$. What we have to show is

(5) $$\;{\left(G⊗E\right)}_{p,q}=\;{\left(Z_{1}{\mathrm{GZ}}_{1}^{t}\;\circ \;Z_{2}{\mathrm{EZ}}_{2}^{t}\right)}_{p,q}$$

Let the design matrices **Z**_1_ and **Z**_2_ be defined as before. Moreover, let the *p*‐th entry of (**ge**) be (ge)_i,j_ and let the *q*‐th entry be ${\left(\mathrm{ge}\right)}_{k,l}$. This means that the *p*‐th row of **Z**_1_ has a 1 at position *i* and the *q*‐th row of **Z**_1_ has a 1 at position *k* (the other entries of these rows are 0). Moreover, the *p*‐th row of **Z**_2_ has a 1 at position *j* and the *q*‐th row of **Z**_2_ has a 1 at position *l* (the other entries of these rows are 0). Thus,

$${\left(Z_{1}{G\;Z}_{1}^{t}\right)}_{p,q}=G_{i,k}\;\mathrm{and}\;{\left(Z_{2}{\mathrm{EZ}}_{2}^{t}\right)}_{p,q}=\;E_{j,l}$$

and consequently

$${\left(Z_{1}{G\;Z}_{1}^{t}\;\circ \;Z_{2}{\mathrm{EZ}}_{2}^{t}\right)}_{p,q}=G_{i,k}\cdot E_{j,l}$$

What remains to be shown is 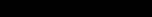. To see this, we use that

(6) $$p=\left(i-1\right)\cdot m+j\;\mathrm{and}\;q=\left(k-1\right)\cdot m+l$$

where *m* is the number of environments (due to the *p*‐th entry of (**ge**) being ${\left(\mathrm{ge}\right)}_{i,j}$ and the *q*‐th entry being ${\left(\mathrm{ge}\right)}_{k,l}$). Moreover, we know that

(7) $$\;{\left(G⊗E\right)}_{p,q}=G_{\left\lceil p/m\right\rceil ,\left\lceil q/m\right\rceil }\cdot E_{p\;\mathrm{mod}\;m,q\;\mathrm{mod}\;m}$$

where ⌈p/m⌉ denotes the least natural number greater than or equal to the ratio p/m (rounding up; ceiling function) and where pmodm denotes the modulo operation, that is division with remainder r and here $r\in \{\begin{array}{c} 1,\ldots ,m\} \end{array}$, not $\left\{ \begin{array}{c} 0,\ldots ,m-1\} \end{array}\right.$. Combining Equations (6) and (7) gives


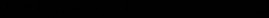




#### 3.1.2 Remark

The setup described above is based on an order of the phenotypes primarily according to line and secondarily according to environment. Of course, this can also be ordered first according to environment and then according to line. In this case, the matrices $\;Z_{1}$ and $Z_{2}\;$will be adapted and the Kronecker product in Equation (4) will change to E⊗G.

The first theoretical result describes the balanced case of having all genotype‐in‐environment combinations in the model and without repetitions. This corresponds exactly to the illustrating example in the Theoretical Background section. We will now go to a more general case in which no restrictions are made on the order of the entries of **y** or on the distribution of lines across environments. Moreover, we can also include repetitions. The second theoretical result is a generalization of the first result.

### 3.2 Second theoretical result

Let **y** be the vector of phenotypes included in the model. An entry of **y** is given by $y_{i,j,rep}$, where *i* indicates the line, *j* the environment the record was taken in, and rep the repetition. Moreover, let ${\tilde{Z}}_{1}$ be a design matrix that maps the corresponding genetic value g_*i*_ to the phenotype $y_{i,j,rep}$, let ${\tilde{Z}}_{2}$ be a matrix that assigns e_*j*_ to $y_{i,j,rep}$, and let ${\tilde{Z}}_{3}$ be a matrix mapping ${\left(\mathrm{ge}\right)}_{i,j}$ to $y_{i,j,rep}$ and which is constructed as described by Bates, Mächler, Bolker, and Walker (2015) using the Khatri‐Rao product

$${\tilde{Z}}_{3}={\left({\tilde{Z}}_{1}^{t}\;*\;{\tilde{Z}}_{2}^{t}\right)}^{t}.$$

Here,(ge) includes all possible genotype‐in‐environment interactions and is sorted first according to genotype and second according to environment.

We consider the model

(8) $$y\;=\;\mu 1_{\left|y\right|}+\;{\tilde{Z}}_{1}g+{\tilde{Z}}_{2}e+{\tilde{Z}}_{3}\;\left(\mathrm{ge}\right)+\;\varepsilon$$

with |y| denoting the length, that is, the number of entries of **y**.

Then

(9) $$\left({\tilde{Z}}_{3}\left(G⊗E\right){\tilde{Z}}_{3}^{t}\right)\;=\;{\tilde{Z}}_{1}{G\tilde{Z}}_{1}^{t}\;\circ \;{\tilde{Z}}_{2}{E\tilde{Z}}_{2}^{t}$$

Which means we can use a Hadamard or a Kronecker formulation to specify the same model.

Before we come to the proof of the second result, please note that Equation (9) is a generalization of Equation (4).

#### 3.2.1 Proof of the second theoretical result

Let the *v*‐th entry of **y** belong to a repetition of line *i* in environment *j* and let the *w*‐th entry of **y** belong to a repetition of line *k* in environment *l*. Then the *v*‐th row of ${\tilde{Z}}_{1}$ has an entry 1 at position *i*. The other entries of this row are equal to 0. Moreover, the *w*‐th row of ${\tilde{Z}}_{1}$ has an entry 1 at position *k*. Analogously, the *v*‐th row of ${\tilde{Z}}_{2}$ has an entry 1 at position *j*, the *w*‐th row of ${\tilde{Z}}_{2}$ has an entry 1 at position *l*. Moreover, using the Khatri‐Rao product definition of ${\tilde{Z}}_{3}$ and the former statements on ${\tilde{Z}}_{1}$ and ${\tilde{Z}}_{2}$, we see that the *v*‐th row of ${\tilde{Z}}_{3}$ has a 1 at position (i−1)·m+j, and the *w*‐th row of ${\tilde{Z}}_{3}$ has a 1 at position (k−1)·m+l.

What we have to show is

$${\left({\tilde{Z}}_{3}\left(G⊗E\right)\;{\tilde{Z}}_{3}^{t}\right)}_{v,w}=\;{\left({\tilde{Z}}_{1}{G\tilde{Z}}_{1}^{t}\;\circ \;{\tilde{Z}}_{2}{E\tilde{Z}}_{2}^{t}\;\right)}_{v,w}$$

First note that according to what we described at which position the rows of the matrices have the entry 1,

$${\left({\tilde{Z}}_{1}{G\;\tilde{Z}}_{1}^{t}\right)}_{v,w}=G_{i,k}\;\mathrm{and}\;{\left({\tilde{Z}}_{2}{E\tilde{Z}}_{2}^{t}\right)}_{v,w}=E_{j,l}$$

Analogously ${\left({\tilde{Z}}_{3}\left(G⊗E\right)\;{\tilde{Z}}_{3}^{t}\right)}_{v,w}$ is the ((i−1)·m+j,(k−1)·m+l) entry of G⊗E.

Using Equation (7) states that this is


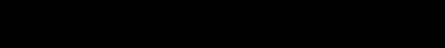

Which means


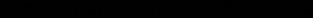




To illustrate a case addressed by the second result, we give a simple example.

### 3.3 A small example for unbalanced data

Let us consider the data

$$y\;={\left(y_{1,1,1},y_{1,1,2},y_{2,2,1},y_{3,2,1},y_{3,3,1},y_{2,2,2}\right)}^{t}$$

consisting of phenotypes of three lines measured in three environments and some with repetitions. The entry y_3, 2, 1_ denotes the first repetition of a record of line 3 grown in environment 2. Note that not all lines have records for each environment and not each combination is repeated. In this example, the design matrices are given by

$$\begin{array}{ccc} {\tilde{Z}}_{1} & = & \left(\begin{array}{ccc} 1 & 0 & 0\\ 1 & 0 & 0\\ 0 & 1 & 0\\ 0 & 0 & 1\\ 0 & 0 & 1\\ 0 & 1 & 0 \end{array}\right),\;{\tilde{Z}}_{2}=\left(\begin{array}{ccc} 1 & 0 & 0\\ 1 & 0 & 0\\ 0 & 1 & 0\\ 0 & 1 & 0\\ 0 & 0 & 1\\ 0 & 1 & 0 \end{array}\right),\\ {\tilde{Z}}_{3} & = & \left(\begin{array}{ccccccccc} 1 & 0 & 0 & 0 & 0 & 0 & 0 & 0 & 0\\ 1 & 0 & 0 & 0 & 0 & 0 & 0 & 0 & 0\\ 0 & 0 & 0 & 0 & 1 & 0 & 0 & 0 & 0\\ 0 & 0 & 0 & 0 & 0 & 0 & 0 & 1 & 0\\ 0 & 0 & 0 & 0 & 0 & 0 & 0 & 0 & 1\\ 0 & 0 & 0 & 0 & 1 & 0 & 0 & 0 & 0 \end{array}\right) \end{array}$$

Use random matrices **G** and **E** to see that Equation (9) is satisfied. Note that ${\tilde{Z}}_{3}={\left({\tilde{Z}}_{1}^{t}*{\tilde{Z}}_{2}^{t}\right)}^{t}\;$and recall that the Khatri‐Rao product is not commutative, that is ${\left({\tilde{Z}}_{1}^{t}*{\tilde{Z}}_{2}^{t}\right)}^{t}\neq {\left({\tilde{Z}}_{2}^{t}*{\tilde{Z}}_{1}^{t}\right)}^{t}$. The order of the multiplication has to be aligned with the order of the Kronecker product on the left‐hand side of Equation (9). If we would use E⊗G instead of G⊗E, the third design matrix would be given by ${\tilde{Z}}_{3}={\left({\tilde{Z}}_{2}^{t}*{\tilde{Z}}_{1}^{t}\right)}^{t}$.

We will now consider a wheat and a maize data set to show that the inclusion of available data from other environments may be beneficial, also in the case that we are only interested in predicting the performance of lines grown in a certain environment.

## 4 MATERIALS AND METHODS

Additionally to the theoretical results, we present some empirical results on predictive ability of models including G × E interaction. In the following, we will explain the data structure and the cross validation scenarios which we used.

### 4.1 Data

#### 4.1.1 Wheat data

We used the wheat data set published by Crossa et al. (2010) which offers genomic data in the form of 1279 presence/absence markers of 599 wheat lines. The phenotypic data provides yield records of these 599 lines grown under four different environmental conditions. The correlation of the phenotypes of the 599 lines grown in the four environments is presented in Table 1. For more information on the data see Crossa et al. (2010).

**TABLE 1:** Phenotypic correlation of the yield records of the 599 lines across the four environments (wheat data)

	E1	E2	E3	E4
**E1**	1	−0.02	−0.19	−0.12
**E2**		1	0.66	0.41
**E3**			1	0.39
**E4**				1

#### 4.1.2 Maize data

We additionally considered a maize data set which has been published by Sousa et al. (2017). The data set USP provides phenotypic records of different traits of 739 lines measured in four environments. We restrict our considerations to yield. Here, 722 lines had records from all four environments, and 17 lines were not observed at each location. The genomic relationship matrix GB was used as provided. The phenotypic correlations of the lines overlapping between each pair of environment are presented in Table 2. For more information on the data, please see Sousa et al. (2017).

**TABLE 2:** Phenotypic correlation of the yield records of the 722 lines planted in all four environments (maize data)

	E1	E2	E3	E4
**E1**	1	0.54	0.33	0.42
**E2**		1	0.37	0.47
**E3**			1	0.49
**E4**				1

### 4.2 Cross validations, statistical models and covariance matrices

To illustrate the properties of G × E models, we considered three different types of cross validations. All predictions were performed using the BGLR package (Perez & de los Campos, 2014).

For the wheat data set, the genomic relationship matrix was constructed as $G:=MM^{t}/p$ (VanRaden, 2008) with Mthe 599 × 1279 marker matrix, and *p* the number of markers (here 1279). For the maize data set, the GBLUP matrix was used as provided by Sousa et al. (2017).

Moreover, when **E** was included in the model, we considered two cases. The first case was E:=I the identity matrix. For the second case, the lines of the training set were used to estimate the pairwise covariances of the phenotypes across different environments and the R function nearPD of the package “Matrix” (Bates and Maechler, 2019) was applied to guarantee the positive‐semi‐definiteness of **E**.

#### 4.2.1 Scenario SC1: Standard within environment CV

The first cross validation was within environment. For the wheat data, a test set of 60 lines was randomly drawn and the genetic values were predicted using the remaining 539 lines. This was repeated 50 times. The average Pearson correlation of predictions and phenotypes of the test set is reported in Table 3. The statistical model used was

$$y\;=\;\mu 1_{599}+\;{\tilde{Z}}_{1}g\;+\;\varepsilon$$

with $g\;∼N(0,\sigma_{g}^{2}\;G)$ and $\varepsilon \;∼N(0,\sigma_{\varepsilon }^{2}\;I)$. The Pearson correlation measured was $\mathrm{cor}(y,{\tilde{Z}}_{1}\hat{g})$ on the test set, that is the set of 60 lines whose genetic values were predicted from the phenotypes of the remaining 539 lines.

**TABLE 3:** Predictive abilities for the wheat data. Mean Pearson correlation of the predicted values and the phenotypes of the test set (60 lines) for the different cross validation scenarios. SC1 is within environment (E1, E2, E3 or E4), ignoring the data from the other environments. SC2 and SC3 use a G × E model and the data of the other environments. The difference between both is whether the phenotypic data of the 60 lines of the test set under different environmental conditions is included (SC2) or not (SC3). Moreover, the E and I indicate whether the identity matrix (I) is used as environmental covariance or whether the environmental covariance is estimated from the phenotypic covariance across environments in the training data (E)

Environment	SC1	SC2‐I	SC2‐E	SC3‐I	SC3‐E
E1	0.517 ± 0.013	0.432 ± 0.016	0.542 ± 0.012	0.461 ± 0.017	0.516 ± 0.013
E2	0.512 ± 0.015	0.562 ± 0.015	0.657 ± 0.010	0.491 ± 0.015	0.532 ± 0.015
E3	0.382 ± 0.014	0.448 ± 0.016	0.528 ± 0.014	0.385 ± 0.017	0.391 ± 0.016
E4	0.468 ± 0.017	0.493 ± 0.012	0.541 ± 0.011	0.453 ± 0.014	0.452 ± 0.013

The maize data set was used analogously, but with a test set of 73 lines.

#### 4.2.2 Scenario SC2: Using data from other environments including the records of the same lines

For the second cross validation, we included the record of the lines from other environment, but the test set is still coming from one environment. For each of the 50 repetitions, 60 lines were drawn as test set from environment *i* and predicted by the data of the remaining 539 lines in environment *i* and the data of all 599 lines in the other three environments. The statistical model used was

$$y\;=\;\mu 1_{599}+\;{\tilde{Z}}_{1}g+{\tilde{Z}}_{2}e+{\tilde{Z}}_{3}\left(\mathrm{ge}\right)+\;\varepsilon$$

with gand ε as before and $e\;∼N(0,\sigma_{e}^{2}\;E)$, $\left(\mathrm{ge}\right)\;∼N(0,\sigma_{ge}^{2}\;G⊗E)$. The design matrices were generated according to the data structure as earlier described. The Pearson correlation measured was $\mathrm{cor}(y,\;{\tilde{Z}}_{1}\hat{g}+{\tilde{Z}}_{3}\left(\hat{\mathrm{ge}}\right))$ for the test set. Since the test set was selected from the same environment, the correlation does not depend on whether the environmental effects are included. Two variants were calculated. The first variant was E:=I. In the second variant, **E** was estimated by the phenotypic correlation across environments of the training set (as described above).

The procedure for the maize data set was analogous, but with a test set of 73 lines.

#### 4.2.3 Scenario SC3: Using data from other environments without the records of the same lines

Scenario SC3 was analogous to SC2, but without using any record of lines in the test set. This means that having drawn a test set in an environment, we used the records of the remaining 539 lines from all four environments as training data.

The procedure for the maize data set was analogous, but with a test set of 73 lines.

## 5 EMPIRICAL RESULTS

### 5.1 Wheat data

The first cross validation scenario SC1 was based on a within environment prediction. In the second scenario SC2, we were again interested in the predictive ability of yield in a particular environment. However, instead of using only the 539 records of the training set of the particular environment, we additionally used the records of the 599 lines in the other three environments. We used a model including G × E effects in two variations. In the first version SC2‐I, we used the identity matrix E:=I. In the second version SC2‐E, we estimated the pairwise phenotypic covariances between environments from the training set. The third cross validation SC3, was the same as SC2, but we did not include the phenotypic data of the lines of the test set grown in the other environments. Instead, we only used the data of the remaining 539 lines in the four environments as training set. The distinction between SC2 and SC3 allows separating the effect of having the same lines under different environmental conditions from the general effect of including G × E and more environments in the training set. We also used the variants SC3‐I and SC3‐E.

The results are summarized in Table 3. Comparing SC1 to SC2‐I, we see that the inclusion of the other environments and modeling G × E increased the predictive ability in environments 2, 3, and 4. However, the predictive ability in environment 1 was drastically reduced (from 0.517 to 0.432). This may be a result of the negative phenotypic correlation, which the phenotypes of environment 1 have with the phenotypes of the other environments (see Table 1, Materials and Methods) and not having included this aspect in the environmental covariance.

Moreover, when we compare SC1 to SC3‐I, we see that the increase in predictive ability given by SC2‐I is lost when the yield performance of the test set lines under different environmental conditions is not included in the training set (compare SC2‐I to SC3‐I). Thus, the main increase of SC2‐I compared to SC1 seems to be a result of having some records of the performance of the test set in the training set. Also the negative impact for the prediction of a test set of environment 1 is reduced when the records of test set lines in other environments are not included in the training set (compare E1, 0.432 for SC2‐I to 0.461 for SC3‐I).

Considering the cross validations SC2‐E and SC3‐E, we again see – as for SC2‐I and SC3‐I and E2, E3, E4 – that SC2‐E leads to higher predictive abilities than SC3‐E. Moreover, when comparing both to their counterpart with the identity matrix, we see that estimating **E** from the training set is beneficial and also improves the prediction of lines in environment 1 (from 0.517 in SC1 to 0.542 in SC2‐E). Comparing SC1 to SC3‐E, the difference in predictive ability is rather small indicating again that a main driver to increase predictive ability is including records of the test set from other environments in the training set.

An important point to highlight is that the predictive ability for SC2‐E was in environment 2 and 3 below the maximal correlation of the phenotypes across environments, which was here 0.66 for environments 2 and 3 (see Table 1). However, for environment 1 and 4, the predictive ability using SC2‐E was above the maximal (absolute) phenotypic correlation with these environments. For instance, E2 has the highest phenotypic correlation with the data of E4 (0.41). The predictive ability for E4 increased from 0.468 (SC1) to 0.541 (SC2‐E). For environments E1 and E4, SC2‐E improved the prediction compared to simply using the phenotypic records from the environment that has the highest correlation with the test environment to predict the test set.

In this context of using the data of the other environments including records of the test set lines, see also the results of Martini et al. (2016). The authors used one environment to identify relevant interactions of an epistasis model and then subnetworks of selected interactions to define a relationship matrix. The latter was subsequently used to predict within another environment. The predictive abilities obtained were very similar to those of SC2‐E, which used the same data basis.

### 5.2 Maize data

The predictive abilities for the maize data set are summarized in Table 4. The patterns observed are similar to those seen on the wheat data set in the sense that SC2‐E is the best scenario for the prediction of three of four environments. An important difference to the results for the wheat data set, is that in none of the cases a predictive ability above the maximal phenotypic correlation with one of the other environments was reached (Table 2). Moreover, in environment 4, the predictive ability of scenario SC3 were a little bit higher than that those of the corresponding SC2 scenarios.

**TABLE 4:** Predictive abilities for the maize data. Mean Pearson correlation of the predicted values and the phenotypes of the test set (73 lines) for the different cross validation scenarios. SC1 is within environment (E1, E2, E3 or E4), ignoring the data from the other environments. SC2 and SC3 use a G × E model and the data of the other environments. The difference between both is whether the phenotypic data of the 73 lines of the test set under different environmental conditions is included (SC2) or not (SC3). Moreover, the E and I indicate whether the identity matrix (I) is used as environmental covariance or whether the environmental covariance is estimated from the phenotypic covariance across environments in the training data (E)

Environment	SC1	SC2‐I	SC2‐E	SC3‐I	SC3‐E
E1	0.278 ± 0.0122	0.313 ± 0.017	0.323 ± 0.017	0.308 ± 0.017	0.308 ± 0.016
E2	0.334 ± 0.014	0.347± 0.017	0.350 ± 0.017	0.342 ± 0.018	0.344 ± 0.018
E3	0.322 ± 0.014	0.367 ± 0.011	0.377 ± 0.011	0.307 ± 0.012	0.307 ± 0.012
E4	0.416 ± 0.014	0.452 ± 0.010	0.455 ± 0.010	0.466 ± 0.010	0.466 ± 0.011

## 6 DISCUSSION

We have shown that the covariance structures of interactions expressed by the Hadamard products $Z_{1}{G\;Z}_{1}^{t}\;\circ \;Z_{2}{\mathrm{EZ}}_{2}^{t}\;$can equally be written as the Kronecker product G⊗E (Equation (4) and with the design matrices $Z_{1},\;Z_{2}\;$as described). An important aspect in this context is the order of the phenotypes. For Equation (4), we used an order of primarily according to the genotypes and secondarily according to the environments. Ordering for instance first according to environments and then according to genotypes would lead to an analogous equation, but with G⊗E commuted to E⊗G in Equation (4). For unbalanced data, when including repetitions, or for an arbitrary order of the phenotypes, the design matrices have to be adapted, but an analogous, more general identity holds (Equation (9)). Thus, if the design matrices are defined according to the order of the phenotypes, both approaches give exactly the same covariance structure.

Having the equivalence of both formulations in mind, it can be interpreted in parts as coincidence that the Hadamard formulation can be found more often in literature addressing G × E, but that the Kronecker formulation has been used for modeling specific combining ability.

### 6.1 Potential computational advantages ‐ Kronecker and Hadamard products and their inverses

Each of the two different presentations may have computational (dis)advantages compared to the other and depending on which mathematical operations should be performed. A classical numerical problem related to the mixed model equations is inverting the covariance matrix. Here, we see that that the Kronecker product may give some advantages since it obeys the identity


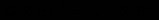

if all matrices are invertible (e.g. Neudecker, 1969). Since the running time of an inversion of a square matrix of size *n* × *n* grows at least as a polynomial of degree 2 (e.g. Petković & Stanimirović, 2009; Wilf, 2002), the right‐hand side is computationally simpler due to the lower dimensionality of the matrices to be inverted. Considering the formulation$\;{\left(Z_{1}{G\;Z}_{1}^{t}\;\circ \;Z_{2}{\mathrm{EZ}}_{2}^{t}\right)}^{-1}$, a similar equality is not given.

### 6.2 The issue of variable coding when modeling interactions by products of predictor variables

We illustrated the equivalence of multiplying entries of the genomic relationship and the environmental covariance, and an approach of using explicitly products of markers and environmental covariables as additional predictors with their own interaction effect (see Appendix). An analogous result has earlier been shown in the context of the interactions within a class of variables, more concrete locus × locus interactions (Jiang & Reif, 2015; Martini et al., 2016). When using products of the independent variables as additional predictors to model interactions between them, the variable coding may have an impact on the prediction outcome when penalized regressions are used (Martini et al., 2017; Martini et al., 2019). Different codings have been discussed, for instance approaching orthogonal estimates of variances (Vitezica et al., 2017). Similar to the locus × locus interaction, the presented equivalences are true for any variable coding (Martini et al., 2016), but the fact that the variable coding will have an impact on the prediction when using a penalized regression method such as RRBLUP/GBLUP equally applies.

### 6.3 Practical implications for sparse testing designs

The results illustrate once more that including G × E effects into prediction models can increase predictive ability (compare to e.g. Burgueño et al., 2012; Cuevas et al., 2016; Jarquin et al., 2014). Concerning the practical application of G × E models, we showed that for the considered methods, the main drivers for the increase in predictive ability are (i) specifying the environmental covariances correctly **and** (ii) having the same lines grown in the other environments (The latter has been highlighted for instance by Burgueño et al., 2012). This becomes evident when considering Tables 3 and 4, for which the scenario SC2‐E had the highest predictive ability for seven out of the eight considered cases. For sparse testing approaches in which the set of lines is partitioned across environments, this means that all lines should be observed in at least one environment but also that the environmental covariance **E** should be determined. If no data on environmental variables is available but there is sufficient overlap of lines between environments, **E** can be estimated as phenotypic (or estimated genetic) covariance.

### 6.4 Outlook

The G × E models have been demonstrated to improve predictive ability in many instances. One important application of these approaches is to define “optimal” sparse testing designs which allow reducing the testing effort without a big loss in accuracy. An investigation of different sparse testing designs together with a comparison of different G × E models may be of interest for the plant breeding community.

## APPENDIX 1

### Products of entries of covariance matrices and explicit interactions

We stated in the main text that we use a model in which

$$\mathrm{COV}\;\left(ge_{ij},ge_{kl}\right)=\;\mathrm{COV}\left(g_{i},g_{k}\right)\cdot \mathrm{COV}\;\left(e_{j},e_{l}\right)=G_{i,k}\cdot E_{j,l}$$

First note that given **A** and **B** are both positive semidefinite, A⊗B and A∘B are both positive semidefinite (if the Hadamard product is defined), which means that these matrix operations give valid covariance structures. A question that may come up is why the product of the entries is a reasonable covariance structure. The matrices **G** and **E** are usually derived from variables such as an *n* × *t* marker data **M** or an *m* × *s* matrix **N** giving environmental variables. A common definition of the covariance structures is then for instance $G:=MM^{t}$ and $E:=NN^{t}$. As before, *n* is the number of lines and *m* is the number of environments. Moreover, *t* and *s* represent the number of variables considered to characterize **G** or **E**, respectively. We can interpret **g** and **e** of Equation (1) as being a result of

$$g:=M\beta_{1}\;\mathrm{and}\;e:=N\beta_{2}$$

with $\beta_{1}$, $\beta_{2}$ independent and$\;\beta_{1}∼N\left(0,\sigma_{\beta_{1}}^{2}\;I\right)$, $\beta_{2}∼N\left(0,\sigma_{\beta_{2}}^{2}\;I\right)$. Moreover, let us model the interaction of two variables by adding an effect for the product of the two variables

$$\begin{array}{ccc} \;y_{i,j} & = & \mu +M_{i,•\;}\beta_{1}+\;N_{j,•\;}\beta_{2}\\ & & +\;\sum_{r=1,\ldots ,t}\;\sum_{u=1,\ldots ,s}M_{i,r}\cdot N_{j,u\;}\cdot \beta_{3,r,u}+\varepsilon_{i,j} \end{array}$$

The term $\sum_{r=1,\ldots ,t;u=1,\ldots ,s}M_{i,r}\cdot N_{j,u}\cdot \beta_{3,r,u}$ with $\beta_{3}∼N\left(0,\sigma_{\beta_{3}}^{2}\;I\right)$ corresponds then to ${\left(\mathrm{ge}\right)}_{i,j}$. We investigate what the induced covariance structure for (**ge)** looks like:

$\mathrm{COV}\;\left(ge_{i,j},ge_{k,l}\right)=\;\mathrm{COV}\left(\sum_{r=1,\ldots ,t;u=1,\ldots ,s}M_{i,r}\cdot N_{j,u\;}\cdot \beta_{3,r,u},\sum_{r=1,\ldots ,t;u=1,\ldots ,s}M_{k,r}\cdot N_{l,u\;}\cdot \beta_{3,r,u}\right)$. Due to the linearity of the COV and the independence of $\beta_{3,r,u}$, this can be written as

$$\begin{array}{ccc} \mathrm{COV}\;\left(ge_{ij},ge_{kl}\right) & = & {\;\sigma }_{\beta_{3}}^{2}\sum_{r=1,\ldots ,t}\;\sum_{u=1,\ldots ,s}M_{i,r}\cdot N_{j,u}\cdot M_{k,r\;}\\ \cdot N_{l,u} & = & {\;\sigma }_{\beta_{3}}^{2}\left(\sum_{r=1,\ldots ,t}M_{i,r}\cdot M_{k,r\;}\right)\\ \cdot \left(\sum_{u=1,\ldots ,s}N_{j,u}\cdot N_{l,u}\right) & = & {\;\sigma }_{\beta_{3}}^{2}G_{i,k}\cdot E_{j,l} \end{array}$$

This illustrates that the product structure of multiplying the covariance matrices element‐wise is equivalent to modeling interactions by products of the underlying variables used to define **G** and **E**. Including products of predictor variables as additional predictors is a common way to model interactions. However, there are other possibilities, for instance modeling an independent effect for each possible category of each pair of markers (e.g. Martini et al., 2017) or other “non‐additive” relationship models such as the Gaussian kernel (e.g. Crossa et al., 2010; de los Campos et al., 2009; Ober et al., 2011).
